# Factors associated with diarrheal morbidity among under-five children in Jigjiga town, Somali Regional State, eastern Ethiopia: a cross-sectional study

**DOI:** 10.1186/s12887-017-0934-5

**Published:** 2017-08-23

**Authors:** Hailemichael Bizuneh, Fentabil Getnet, Beyene Meressa, Yonatan Tegene, Getnet Worku

**Affiliations:** 1 Epidemiology and Biostatistics Unit, Department of Public Health, College of Medicine and Health Sciences, Jigjiga University, Jigjiga, Somali Regional State Ethiopia; 2 School of Graduate Studies, College of Medicine and Health Sciences, Jigjiga University, Jigjiga, Somali Regional State Ethiopia; 3 Department of Nursing, College of Medicine and Health Sciences, Jigjiga University, Jigjiga, Somali Regional State Ethiopia

**Keywords:** Underfive, Diarrhea, Factors, Hygiene, Jigjiga, Ethiopia

## Abstract

**Background:**

The prevalence of underfive diarrhea in Somali Regional State, Ethiopia is one of the highest in the country. This study attempted to examine the multiple factors associated with underfive diarrhea and how they might influence its prevalence in Jigjiga, Somali regional state, Ethiopia.

**Methods:**

A community based cross-sectional study was conducted from February 15 to 28, 2015. Multistage sampling technique was used to collect data from 492 mothers via household survey. A pre-tested, structured questionnaire was used to collect data through face-to-face interview. Ethical clearance was obtained before data collection. Stepwise multivariable logistic regression was used to calculate adjusted odds ratios.

**Results:**

The two weeks prevalence of under five diarrhea in Jigjiga town was 14.6%. Up on multivariable analysis, maternal educational level of primary school and above was found to be protective against childhood diarrhea [AOR: 0.227(0.100–0.517)] whereas, unavailability of water [AOR: 2.124(1.231–3.664)] and lack of hand washing facility [AOR: 1.846(1.013–3.362)] were associated with diarrhea.

**Conclusion:**

Poor water supply, lack of hand washing facilities and lack of formal maternal education were associated with underfive diarrhea in the study area. Improved access to water supply along with environmental health intervention programs designed to promote good hygiene behavior could be of paramount importance to alleviate burden of childhood diarrhea.

**Electronic supplementary material:**

The online version of this article (doi:10.1186/s12887-017-0934-5) contains supplementary material, which is available to authorized users.

## Background

Diarrhea is one of the leading infectious causes of morbidity and mortality in underfive children [1, 2]. In 2015, it was estimated that 1.3 million deaths were caused by diarrheal diseases, of which 499,000 were underfive children [3]. Eventhough decline in diarrheal diseases is reported [4] Sub-sahran Africa, including Ethiopia, remains one of the highly affected regions [5–8].

The underfive mortality rate in Ethiopia, 88 per 1000, indicates that the effort towards reduction of underfive mortality in Ethiopia is still a long way ahead if the country is to achieve the Millennium Development Goal 4 target of reducing the underfive mortality rate by two-thirds between 1990 and 2015. In 2010, in Ethiopia, diarrhea was responsible for 8% of all under-5 mortality and 13% of morbidity amongst under five children. The proportion of children with diarrhea for whom advice or treatment was sought from a health care provider was only 32% [9, 10].

According to Ethiopian Demographic and Health Survey data for under-5 mortality, Somali regional state reported 93 deaths per 1000 live births and two weeks prevalence of diarrhea was 19.5%, which is the third highest in the country next to Gambella and Benishangul regional states. In Somali region, 54.4% of underfive children did not receive any form of treatment for diarrheal morbidity [10].

In Ethiopia, various strategies have been implemented to reduce child mortality from diarrhea. The Health Sector Development Program IV (2010/11–2014/15), which is a policy implementation strategic document that guides the development of sub national plans and sets the rule of engagement in the health sector, aimed to decrease under-five mortality rate from 101/1000 live births to 68/1000. In doing so, the Integrated Management of Childhood Illness approach, has been implemented as a major strategy. Despite the effort, underfive child mortality in the country remains one of the highest in sub-sahran Africa [11, 12].

Sociodemographic, household, environmental, and host characteristics play an important role in determining risk and recovery from diarrheal episodes. Many of the risk factors for contracting diarrheal illnesses are associated with poor socioeconomic conditions such as: lacking access to safe water and sanitation, poor hygiene practices, unsafe human waste disposal, limited access to health care, education, poor diet and housing conditions [2, 8, 13].

So far, few studies have been conducted to identify factors associated with underfive diarrhea in Ethiopia, but a study conducted in Somali Regional State and specifically Jigjiga town is lacking. As Somali region has the third highest underfive diarrheal morbidity and mortality rate in the country, a study which addresses the topic is much needed. This study attempted to examine the prevalence and factors associated with underfive diarrhea in Jigjiga, Ethiopia.

## Methods

This study was a community based cross-sectional study conducted from February 15–28, 2015. Jigjiga is the capital town of Somali Regional State located in the eastern part of Ethiopia. The population of the town is estimated to be 159,300. The population is mainly of Somali extraction and most residents are Muslim. There is one referral and one zonal hospital, and two health centers in town.

Sample size was computed based on single population proportion formula assuming 95% confidence interval, 5% margin of error, prevalence (P) of 19.5% (two weeks prevalence of diarrhea among children under age five in Somali Regional State, Ethiopia) [10], a design effect of 2 (since multistage cluster sampling was used) and a non-response rate of 10% which gave a final sample size of 530.

A multistage sampling technique was used including *Kebeles* (administrative sub divisions of town) as first-stage units, and *Ketenas* (non-administrative sub divisions of *kebeles*) as second-stage units, and households as third-stage units. First, of the total 10 *kebeles* in the town, 5 kebeles were selected by lottery method. Then, *ketenas* were chosen from each of the 5 kebeles. The number of *ketenas* included was proportionally allocated to the size of the *kebeles*. Since the number of underfive children residing in the selected *ketenas* was not available, a census was conducted in all the selected *ketenas* to have a sampling frame. Based on this sampling frame obtained from the census, the final sample size of 530 was proportionally allocated among the *ketenas. S*ystematic random sampling was used within each *ketenas* to select households for interview. In households with more than one under five index children or more than one mother or caregiver, lottery method was used to choose one. Two revisits to a household were made for respondents unavailable at the time of data collection. Inclusion criteria for the study participants were being an index underfive child, and a mother or care giver who is permanent resident of the town.

### Operational definitions

- **Diarrhea** – the presence three and more loose or liquid stools per day within two weeks period prior to survey
- **Dirt floor**: floor made of earth, sand or dung
- **Non-dirt floor** – floor made of wood planks, palm/bamboo, parquet/polished, vinyl or asphalt strips, ceramic tiles or cement
- **Hand washing**: the physical removal of microorganisms from the hands using soap (plain or antimicrobial) and running water.
- **Improved water source**: piped water, piped water to yard/plot, public tap or stand pipe, protected dug well and bottled water
- **Unimproved water sources**: unprotected dug well, tanker-truck, surface water (river, pond and stream)
- **Proper solid waste disposal** – burying or storing in a container and disposing in designed site
- **Improper solid waste disposal** – burning, open field disposal
- **Improved liquid waste disposal** – flush toilet, flush/pour to pit latrine, ventilated improved pit latrine and pit latrine with slab
- **Unimproved liquid waste disposal** – flush to elsewhere, pit latrine without slab, bucket and no facility
- **Hand washing at critical times** – washing hands before and after cooking foods, after latrine use, and before feeding child

The outcome variable was diarrhea and independent variables were composed of socio demographic variables, household and environmental, hygiene behavior and child feeding practices. A questionnaire adapted from World Health Organization (Core questions on drinking water and sanitation for household surveys) [14] composed of closed-ended questions was used in preparation of the instrument. The final questionnaire was translated to *Somali* language and back to English language to check for consistency. The questionnaire was pretested on 5% of the total sample size, i.e. 27 mothers of underfive children who resided in a *Kebele* outside the study area. Finally a pretested, structured questionnaire was used to conduct face-to-face interviews Additional file 1. Data collectors proficient in the local *Somali* language were trained by the researchers. On-field supervision of data collectors was carried out.

Data was entered, cleaned and analyzed using Statistical Package for Social Sciences. Descriptive statistics was used to present results. Crude and adjusted odds ratio with 95% confidence interval were also calculated in univariate and multivariable logistic regressions. In order to identify independent factors associated with the outcome, variables significantly associated on univariate analysis at a cut off point *p* value 0.3 were put in to multivariable model for further analysis. Backward stepwise regression was implemented to identify final adjusted odds ratios of independent factors associated with diarrhea at a cut-off point *p*-value 0.05.

### Ethical consideration

All caregivers underwent informed consent for participation in the study. Ethical clearance was obtained from the Institutional Review Board of Jigjiga University, Directorate of Research, Publication and Technology Transfer.

## Results

Out of the total of 530 mothers and underfive children targeted for the study, 492 mothers were interviewed. The mean age of mothers was 28.6 (±6.57) years, and 146 (29.7%) mothers had no formal education. Close to one-half (52.6%) of the households had a family size fewer than five with a mean of 5 (±2.06**).** Almost all (97.6%) of households used protected water sources such as public standpipes. Hand washing stand was not present in most (77.2%) of the households**.** In 117 (23.8%) of the households water was drawn from storage containers by dipping. In most (64.5%) of households latrines were shared with one or more neighbors. Out of which, 141 (44.3%) were shared with more than five households. Majority (94.3%) of the toilet facilities were improved. The mean age of index children was 22.53 (±1.48) months. Most (65.3%) of children were currently breast feeding, and 443 (90.4%) started complementary food after the age of six month. Hand washing at critical time was self-reported by 330 (67.1%) of mothers. The two weeks prevalence of diarrhea was 14.6% [Table 1].

**Table 1:** Characteristics of children under age five, Jigjiga town, 2015

Variable	Frequency	Percent
Primary care giver’s characteristics		
Age		
15–24	146	29.6
25–34	234	47.6
35–49	112	22.8
Educational status		
No education	146	29.7
Primary and above	346	70.3
Household size		
< 5	259	52.6
5 and above	233	47.4
Number of underfive children		
One	358	72.8
Two and above	134	27.2
Child characteristics		
Age of index child		
< 6	49	10
6–11	81	16.5
12–23	125	25.4
> 23	237	48.1
Sex		
Male	250	50.8
Female	242	49.2
Environmental characteristics		
Floor type		
Dirt	104	21.1
Not dirt	388	78.9
Source of water		
Protected	158	32.1
Unprotected	334	67.9
Water available all the time		
Yes	244	49.6
No	248	50.4
Method of water drawing		
Dipping	117	23.8
Pouring	375	76.2
Hand washing facility present		
Yes	112	22.8
No	380	77.2
Latrine availability		
Yes	478	97.1
No	14	2.9
Ownership of latrine		
Private	174	35.5
Shared	318	64.5
Shared with how many households (*n* = 318)		
< 5	177	55.7
> 5	141	44.3
Toilet facility		
Improved	479	97.3
Non-improved	13	2.7
Hand washing at critical times		
High	330	67.1
Low	162	32.9
Feeding characteristics		
Currently breast feeding		
Yes	321	65.3
No	171	34.7
Age complementary food started		
< 6 (Not yet started)	41	8.3
Before 6 month	8	1.6
After 6 month	443	90.4
Diarrhea in past two weeks		
Yes	72	14.6
No	420	85.4

### Factors associated with underfive diarrhea

Main variables which were significantly associated with underfive diarrhea upon univariate analysis were: lack of maternal education [COR: 0.227(0.100–0.517)], bigger family size [COR: 1.719(1.025–2.885)], lack of water [COR: 2.128 (1.263–3.585)], lack of hand washing stand [COR: 2.195(1.284–3.752)], latrine sharing [COR: 0.548(0.305–0.987)], drawing water by dipping [COR: 2.119(1.049–4.281)], child age groups 6–11 month [COR: 0.184(0.095–0.357)] and 12–23 months [COR: 0.408(0.212–0.787)]. After backward stepwise multivariable regression, variables significantly associated with the outcome variable were: lack of formal education of mothers, unavailability of water, and lack of hand washing facility. Children of mothers with primary education and above were less likely to have diarrhea [AOR: 0.227(0.100–0.517)]. In houses where water was not available all the time, the odds of diarrhea among children was doubled [AOR: 2.124(1.231–3.664)]. Presence of hand washing stand was a significant predictor as children in households with no hand washing stand were 1.846 times more likely to have diarrhea [AOR: 1.846(1.013–3.362)] [Table 2].

**Table 2:** Multivariable analysis results of factors associated with diarrhea among underfive children in Jigjiga town, February, 2015

Variables	Diarrhea		Crude OR	Adjusted OR	*P* value
	Yes	No			
Maternal age					
15–24	22	124	0.676(0.319–1.433)		
25–34	39	195	0.600(0.301–1.197)		
35–49	12	100	1.00		
Maternal education					
No education	13	133	1.00	1.00	0.000
Primary and above	59	287	0.475(0.252–0.897)*	0.227(0.100–0.517)*	
Child age					
< 6	6	43	0.625(0.236–1.655)		
6–11	26	55	0.184(0.095–0.357)*		
12–23	22	103	0.408(0.212–0.787)*		
> 23	19	218	1.00		
Number of underfive children					
One	50	308	1.00		
Two and above	23	111	0.783(0.457–1.344)		
Family size					
< 5	46	213	1.00		
> 5	26	207	1.719(1.025–2.885)*		
Water availability					
Yes	47	197	1.00	1.00	0.007
No	25	223	2.128(1.263–3.585)*	2.124(1.231–3.664)*	
Method of drawing water					
Dipping	10	107	2.119(1.049–4.281)*		
Pouring	62	313	1.00		
Hand washing facility					
Yes	26	86	1.00	1.00	
No	46	334	2.195(1.284–3.752)*	1.846(1.013–3.362)*	0.045
Toilet facility					
Improved	70	409	1.00		
Non-improved	3	10	0.570(0.153–2.125)		
Latrine shared					
< 5	41	136	0.548(0.305–0.987)*		
> 5	20	121	1.00		

*P value <0.05

## Discussion

This study identified socio-demographic and environmental factors associated with diarrhea among underfive children in the study area. Diarrhea was significantly associated with lack of formal maternal education, poor water supply and lack of hand washing facilities.

In this study, two weeks prevalence of diarrhea among underfive children was 14.6%. This prevalence is relatively lower as compared to findings of studies done in other parts of Ethiopia which reported higher prevalences such as: Nekemte (28.9%), Eastern Ethiopia (22.5%), Sheko district (25%), Gilgel Gibe research centre (30%) and West Gojam (18%) [15–19]. This might be attributed to differences in socio demographic and environmental factors. As the studies were conducted in various parts of the country, important factors such as provision basic hygienic services which might strongly affect underfive diarrhea differ across settings. For instance, the latrine coverage in Nekemte town (91.8%) [15] is lower than that of Jigjiga’s (97.1%) and hand washing at critical times in Sheko was reported to be 61.5% [17] which is also lower than that of Jigjiga’s (67.1%).

Among socio-demographic variables studied, maternal education was found to be an independent predictor of diarrhea. Children of educated mothers were protected against diarrhea as compared to children of mothers with no formal education. This finding is consistent with studies conducted in Ethiopia, Uganda and Afghanistan where mothers’ education, especially at post-secondary level, reduced the probability of occurrence of diarrhea [17–22]. This might be due to the fact that mothers who had formal education are prone to have more awareness on good hygiene behaviours, safe handling of water, point-of-use treatment, safe disposal of domestic waste, and good child feeding practices.

Studies have shown that household and environmental factors contribute to childhood diarrhea in Ethiopia. Main factors include: lack of latrine, improper child stool disposal, improper refuse disposal, poor handling of water in households and lack of hand washing facilities [17, 18, 22–24]. Similarly, in Jigjiga town, children living in households where water was not available were found to be at higher risk of having diarrhea. This is also in line with a studies conducted in low and middle income countries [7].

Quantity of water and convenience of the source are more important than quality of water for reducing diarrheal illnesses [7]. In this study area, most of the community primarily uses water from communal stand pipes. The water is delivered to households by plastic barrels pulled by donkey carts. Moreover, water sources in the town are often intermittent leaving the community without water and forced to use alternative unprotected sources. This lack of accessible and consistent water supply could serve as a disabling factor for mothers’ safe hygiene practices which could explain the higher prevalence of diarrhea in households without water.

In this study, lack of hand washing stand was a significant factor to predict diarrhea among under five children. This finding is similar to a study conducted in Eastern Ethiopia which reported a significant positive association between the availability of hand washing facility and childhood diarrhea [16]. In the current study, lack of consistent source of water supply could have served as a factor that exacerbated lack of hand washing facilities which in turn predicted diarrhea.

Possible limitations of the study include that it did not comprehensively address the possible factors associated with underfive diarrhea. Hygiene behaviour was assessed based on self report rather than observation. Self-report of diarrhea over a two week period could have underestimated true magnitude. It is also impossible to know if some risk factors preceded the outcome or changed because of it. In addition, due to lack of data on the distribution of pathogens among the children with diarrhea, the generalizability of the identified factors may apply only to other settings where the pathogen distribution is similar to the one in this study.

## Conclusion

The study has mainly identified socio demographic and environmental factors associated with diarrhea among underfive children. The two week prevalence of diarrhea was found to be relatively low in the study area. Lower maternal educational level, unavailability of water and lack of hand washing facility were important factors associated with diarrhea. Provision of improved access to water supply to the community is important in tackling use of alternative unprotected water sources. Supportive supervision of households to construct hand washing stands using locally available materials and point-of-use water treatment methods can also help alleviate the problem. In addition, environmental health intervention program designed to promote mothers’ good hygiene behavior, safe handling of water and safe disposal of waste is crucial.

## Additional file

Additional file 1: Data collection tool. (DOC 82 kb)

## References

[1] UNICEF (2012). Committing to child survival: a promise renewed, a global effort to accelerate action on maternal, newborn and child survival.

[2] Fischer Walker CL, Perin J, Aryee MJ, Boschi-Pinto C, Black RE (2012). Diarrhea incidence in low- and middle-income countries in 1990 and 2010: a systematic review. BMC Public Health.

[3] Wang H (2016). Global, regional, and national life expectancy, all-cause mortality, and cause-specific mortality for 249 causes of death, 1980–2015: a systematic analysis for the Global Burden of Disease Study 2015. Lancet.

[4] Liu L (2015). Global, regional, and national causes of child mortality in 2000–13, with projections to inform post-2015 priorities: an updated systematic analysis. Lancet.

[5] Boschi-Pinto C, Velebit L, Shibuya K (2008). Estimating child mortality due to diarrhoea in developing countries. Bull World Health Organ.

[6] UNICEF/WHO (2009). Diarrhea: why children are still dying and what can be done.

[7] UNICEF (2012). Pneumonia and diarrhea: tackling the deadliest diseases for the world’s poorest children.

[8] WHO/Unicef (2013). Ending preventable deaths from Pneumonia and diarrhea by 2025; the integrated action Plan for prevention and diarrhea.

[9] United nations (2005). The millennium development goals report.

[10] Central Statistical Agency (2012). ICF international Ethiopia demographic health survey 2011.

[11] Federal Ministry of Health of Ethiopia (2010). Health Sector Development Program IV.

[12] Federal Ministry of Health of Ethiopia (2005). National strategy for child survival in Ethiopia.

[13] Carvajal-Vélez (2016). Diarrhea management in children under five in sub-Saharan Africa: does the source of care matter? A Countdown analysis. BMC Public Health.

[14] WHO/UNICEF (2006). Core questions on drinking water and sanitation for household surveys.

[15] Eshete WB (2008). A stepwise regression analysis on under-five diarrhoael morbidity prevalence in Nekemte town, western Ethiopia: maternal care giving and hygiene behavioral determinants. East Afr J Public Health.

[16] Mengistie B, Berhane Y, Worku A (2013). Household water chlorination reduces incidence od diharrea among under-five children in rural Ethiopia: a cluster randomized controlled trial. PLoS One.

[17] Gebru T, Taha M, Kassahun W (2014). Risk factors of diarrhoeal disease in under-five children among health extension model and non-model families in Sheko district rural community, Southwest Ethiopia: comparative cross-sectional study. BMC Public Health.

[18] Amare D, Fasil T, Belaineh G (2007). Determinants of under-five mortality in Gilgel Gibe Field Research Center. Ethiop J Health Dev.

[19] Dessalegn M, Kumie A, Tefera W. Predictors of under-five childhood diarrhea: Mecha District, West Gojam, Ethiopia. Ethiopian journal of health development. 2011;25(3):192–200.

[20] Aluisio AR (2015). Risk factors associated with recurrent diarrheal illnesses among children in Kabul, Afghanistan: a prospective cohort study. PLoS One.

[21] Bbaale E (2011). Determinants of diarrhoea and acute respiratory infection among under-fives in Uganda. Australas Med J.

[22] Sinmegn Mihrete T, Asres Alemie G, Shimeka TA (2014). Determinants of childhood diarrhea among underfive children in Benishangul Gumuz Regional State, North West Ethiopia. BMC Pediatr.

[23] Anteneh ZA, Andargie K, Tarekegn M (2017). Prevalence and determinants of acute diarrhea among children younger than five years old in Jabithennan District, Northwest Ethiopia, 2014. BMC Public Health.

[24] Fekadeselassie B, Yemane B (2014). Under five diarrhea among model household and non model households in Hawassa, South Ethiopia: a comparative cross-sectional community based survey. Bio Med Central Public Health.

