# Supplementary material for: Factors associated with diarrheal morbidity among under-five children in Jigjiga town, Somali Regional State, eastern Ethiopia: a cross-sectional study
Source: BMC Pediatr. 2017 Aug 23;17:182. doi: 10.1186/s12887-017-0934-5 (PMC5568275; doi:10.1186/s12887-017-0934-5)
Supplement: Additional file 1: — Data collection tool. (DOC 82 kb) [file 12887_2017_934_MOESM1_ESM.doc]

Additional file 1 - Data collection tool

| **Part One - Socio-demographic characteristics** | | | |
| --- | --- | --- | --- |
| **No** | **Questions** | **Alternative choices** | **Skip to** |
| 1.1 | How many family members do you have? |  |  |
| 1.2 | How many under five children do you have? |  |  |
| 1.3 | How old are you? |  |  |
| 1.4 | What is your religion? | 1. Orthodox 2. Muslim 3. Catholic 4. Protestant 5. Other (specify)________ |  |
| 1.5 | What is your educational level? | 1. Cannot read and write  2. Read and write only  3. Primary complete  4. Secondary complete  5. College and above |  |
| 1.6 | What is your occupational status? | 1. Civil servant  2. Private employee  3. Student  4. House wife  5. Other(specify) ________ |  |
| 1.7 | What is your ethnicity? | 1. Somali 2. Oromo 3. Tigrie 4. Gurage   5. Amhara  6. Others(specify)________ |  |

| **Part two – Environmental factors – Household factors and Water supply** | | | |
| --- | --- | --- | --- |
| **No** | **Questions** | **Alternative choices** | **Skip to** |
| 2.1 | Type of floor material of the living house | 1. Dirt 2. Cement 3. Wood 4. Other (specify)_________ |  |
| 2.2 | Type of drinking water sources | 1. Piped water into dwelling 2. Public tap or standpipe 3. Well 4. River 5. Other (specify) _________ |  |
| 2.3 | Is water available all the time? | 1. Yes 2. No |  |
| 2.4 | Does the water storage container has cover? | 1. Yes  2. No |  |
| 2.5 | Method of drawing of water from the storage container | 1. Pouring 2. Dipping |  |
| 2.6 | Is there a separate can for taking drinking water from the storage container? | 1. Yes 2. No |  |

| **Part three – Environmental factors – Waste disposal** | | | |
| --- | --- | --- | --- |
| **No** | **Questions** | **Alternative choices** | **Skip to** |
| 3.1 | Do you have latrine facility? | 1. Yes 2. No | If yes, skip to 3.3. |
| 3.2 | If there is no latrine, where do you dispose child’s waste? | 1. Open field 2. Water drainage 3. Other (specify)_______ |  |
| 3.3 | Ownership of the latrine | 1. Privately owned 2. Shared with neighbors |  |
| 3.4 | If shared, how many other households use the latrine? |  |  |
| 3.5 | Is feces seen around the pit-hole or on the floor? | 1. Yes 2. No |  |
| 3.6 | Is feces seen around the house or in the compound? | 1. Yes 2. No |  |
| 3.7 | Type of latrine facility | 1. Unimproved traditional pit latrine 2. Improved traditional pit latrine 3. Ventilated improved pit latrine 4. Flush type 5. Other (specify) ________ |  |
| 3.8 | Is the latrine currently functioning? | 1. Yes 2. No |  |
| 3.9 | How do you dispose refuse? | 1. Pit 2. Burning 3. Open field 4. Garbage cans 5. Other specify____ |  |
| 3.10 | Is there a hand washing facility near latrine? | 1. Yes 2. No |  |

| **Part four – Hygiene Behavior of mother** | | | |
| --- | --- | --- | --- |
| **No** | **Questions** | **Alternative choices** | **Skip to** |
| 4.1 | Do you always wash your hands before food preparation? | 1. Yes 2. No |  |
| 4.2 | Do you always wash your hands before feeding child? | 1. Yes 2. No |  |
| 4.3 | Do you always wash your hands after visiting latrine | 1. Yes 2. No |  |
| 4.4 | Do you always wash your hands after handling child feces? | 1. Yes 2. No |  |
| 4.5 | What did you use to wash your hands yesterday? | 1. Only water 2. Soap and water 3. Other (specify)_______ |  |

| **Part five – Index child characteristics and feeding** | | | |
| --- | --- | --- | --- |
| **No** | **Questions** | **Alternative choices** | **Skip to** |
| 5.1 | Age (In months) |  |  |
| 5.2 | Sex |  |  |
| 5.3 | Birth order |  |  |
| 5.4 | Have you ever breast fed your child? | 1. Yes 2. No | If no, skip to 5.7 |
| 5.5 | For how long did you breastfed your child? |  |  |
| 5.6 | What is his/her current breast feeding status? | 1. Exclusive 2. Partial 3. Not breastfeeding |  |
| 5.7 | At what age the child started supplementary food? (In months) |  |  |
| 5.8 | Did your child have diharrea in the past two weeks? | 1. Yes 2. No |  |
